# The lipolysis inhibitor acipimox reverses the cardiac phenotype induced by electronic cigarettes

**DOI:** 10.1038/s41598-023-44082-x

**Published:** 2023-10-25

**Authors:** Jorge Espinoza-Derout, Jose Mari Luis Arambulo, William Ramirez-Trillo, Juan Carlos Rivera, Kamrul M. Hasan, Candice J. Lao, Maria C. Jordan, Xuesi M. Shao, Kenneth P. Roos, Amiya P. Sinha-Hikim, Theodore C. Friedman

**Affiliations:** 1 Division of Endocrinology, Metabolism and Molecular Medicine, Department of Internal Medicine, Charles R. Drew University of Medicine and Science, 1731 E. 120th Street, Los Angeles, CA 90059 USA; 2 Departments of Physiology, Medicine, and Neurobiology, David Geffen School of Medicine at University of California, Los Angeles, Los Angeles, CA 90095 USA

**Keywords:** Biochemistry, Cell biology, Cardiology, Cardiovascular biology

## Abstract

Electronic cigarettes (e-cigarettes) are a prevalent alternative to conventional nicotine cigarettes among smokers and people who have never smoked. Increased concentrations of serum free fatty acids (FFAs) are crucial in generating lipotoxicity. We studied the effects of acipimox, an antilipolytic drug, on e-cigarette-induced cardiac dysfunction. C57BL/6J wild-type mice on high fat diet were treated with saline, e-cigarette with 2.4% nicotine [e-cigarette (2.4%)], and e-cigarette (2.4%) plus acipimox for 12 weeks. Fractional shortening and ejection fraction were diminished in mice exposed to e-cigarettes (2.4%) compared with saline and acipimox-treated mice. Mice exposed to e-cigarette (2.4%) had increased circulating levels of inflammatory cytokines and FFAs, which were diminished by acipimox. Gene Set Enrichment Analysis revealed that e-cigarette (2.4%)-treated mice had gene expression changes in the G2/M DNA damage checkpoint pathway that was normalized by acipimox. Accordingly, we showed that acipimox suppressed the nuclear localization of phospho-p53 induced by e-cigarette (2.4%). Additionally, e-cigarette (2.4%) increased the apurinic/apyrimidinic sites, a marker of oxidative DNA damage which was normalized by acipimox. Mice exposed to e-cigarette (2.4%) had increased cardiac Heme oxygenase 1 protein levels and 4-hydroxynonenal (4-HNE). These markers of oxidative stress were decreased by acipimox. Therefore, inhibiting lipolysis with acipimox normalizes the physiological changes induced by e-cigarettes and the associated increase in inflammatory cytokines, oxidative stress, and DNA damage.

## Introduction

Electronic cigarettes (e-cigarettes) are battery-powered appliances that generate an aerosol by heating a solution containing nicotine, glycerol, propylene glycol, and flavors^1^. Delivering nicotine to users without burning tobacco, e-cigarettes became the most frequently used tobacco product among U.S. adolescents^2^. In addition, e-cigarettes have become progressively attractive to adults^3^. The detrimental effects of e-cigarettes on the heart have been reported in rodent models and humans^1,4^. Therefore, there is a pressing need for a mechanistic understanding of the cardiac effects of e-cigarettes.

Free fatty acids (FFAs) released by adipose tissue are critical elements in ectopic lipid accumulation, lipotoxicity, mitochondrial dysfunction, and cardiomyopathy^5^. Nicotine-induced sympathetic activation led to lipolysis and increased serum levels of FFAs^6^. Systemic increase of nicotine produces the release of catecholamines that bind to β-adrenergic receptors found in adipocytes, leading to lipolysis^1^. Also, in adipocytes, the activation of nicotinic acetylcholine receptors (nAChRs) by nicotine produces AMPK activation and the release of FFAs^7^. Increased FFAs led to an inflammatory state characterized by infiltration and expansion of lymphocytes and macrophages, which produce proinflammatory cytokines^8^. The nicotine modulation of cytokines in smokers compared to non-smokers may have a profound impact on the cardiovascular system^9–11^.

FFAs can increase mitochondrial generation of reactive oxygen species (ROS), which have been proposed as a significant mechanism of cardiomyopathy and metabolic syndrome^5^. Accordingly, e-cigarettes produce increased oxidative biomarkers and sympathetic dominance in humans^12^ and mouse models^13^. Increased ROS can lead to activation of proapoptotic signaling resulting in cardiac remodeling and mitochondrial dysfunction^6^. Mitochondria is a crucial source of ROS and also one of the main targets of ROS damage. DNA damage produced by oxidative stress has a solid mechanistic link to the pathophysiology of metabolic disease^14^. E-cigarettes induce DNA damage in the lungs, and hearts of mice^15^. Also, the liver of mice exposed to e-cigarettes has increased apurinic/apyrimidinic (AP) sites, indicating DNA damage^16^.

Acipimox is a nicotinic acid analog that binds to hydroxycarboxylic acid receptor 2 (HCA2/GPR109) receptor^17^. HCA2 receptors are highly expressed in adipose tissue^17^. The activation of HCA2 receptors in adipose tissue leads to inhibition of adenylate cyclase and a decreased cAMP response, followed by the suppression of adipocyte triglyceride lipase (ATGL)^18^. In adipocytes, the rate-limiting enzyme for lipolytic activity is ATGL. In addition, inhibition of lipolysis with acipimox normalized the hepatic metabolic changes induced by the treatment with high-fat diet (HFD) plus nicotine^19^.

In the right ventricle, nicotine treatment leads to a7nAChR activation and fibroblast proliferation, collagen production, and extracellular matrix remodeling^11^. In a mouse model of systemic hypertension, nicotine supplied via mini osmotic pump can increase angiotensin II-induced cardiovascular remodeling^20^. In a nicotine-dependent manner, e-cigarette treatment for 12 weeks led to cardiac dysfunction and atherosclerosis in ApoE knockout mouse model^4^. This cardiac phenotype was associated with increased ultrastructural abnormalities indicative of cardiac dysfunction and MDA generation, a marker of oxidative stress^4^. The ventricular transcriptomic analysis exposed changes in genes associated with metabolism, circadian rhythm, and inflammation in e-cigarette-exposed mice^4^. In C57BL/6 wild-type mice on HFD, echocardiographic data showed that mice treated with e-cigarettes had decreased left ventricular fractional shortening (LV%FS) and ejection fraction compared to controls associated with an inflammatory phenotype in e-cigarette-treated mice^19^. This nicotine-dependent phenotype was associated with increased plasma levels of FFAs and oxidative stress, without cardiac hypertrophy^19^. A comparable phenotype produced by e-cigarettes in rats reported fibrosis, oxidative stress, and inflammation but with cardiac hypertrophy^21^.

In developed countries, obesity is highly prevalent among the adolescent and adult populations. Importantly, the combination of smoking and obesity results in increased mortality risk^22^. Obese individuals have a higher probability of using e-cigarettes^23^. The C57BL/6 mouse in HFD model is widely used for studies of diet-induced obesity and its cardiac complications. Non-obese C57BL/6 mice in normal chow diet (NCD) mice have been exposed to e-cigarettes for eight months without showing a significant change in LV%FS or ejection fraction^13^. The induction of cardiac dysfunction by e-cigarettes has been characterized in C57BL/6 mice fed a HFD^19^. Therefore, we used this diet-induced obesity model to study the role of lipolysis inhibition with acipimox in e-cigarette-induced cardiac dysfunction.

## Results

Eight-week-old wild-type mice on a HFD were exposed to saline aerosol, e-cigarettes with nicotine (2.4%) [e-cigarette (2.4%)], e-cigarette (2.4%) plus acipimox [e-cigarette (2.4%) + ACIP] for 12 weeks as described before^4,16,19^. In this model, we have described the levels of nicotine and cotinine in the plasma measured by LC–MS/MS are in ranges similar to those found in habitual smokers^19^.

### Echocardiographic assessment of acipimox effects on e-cigarette-induced cardiac dysfunction

**Figure 1:**
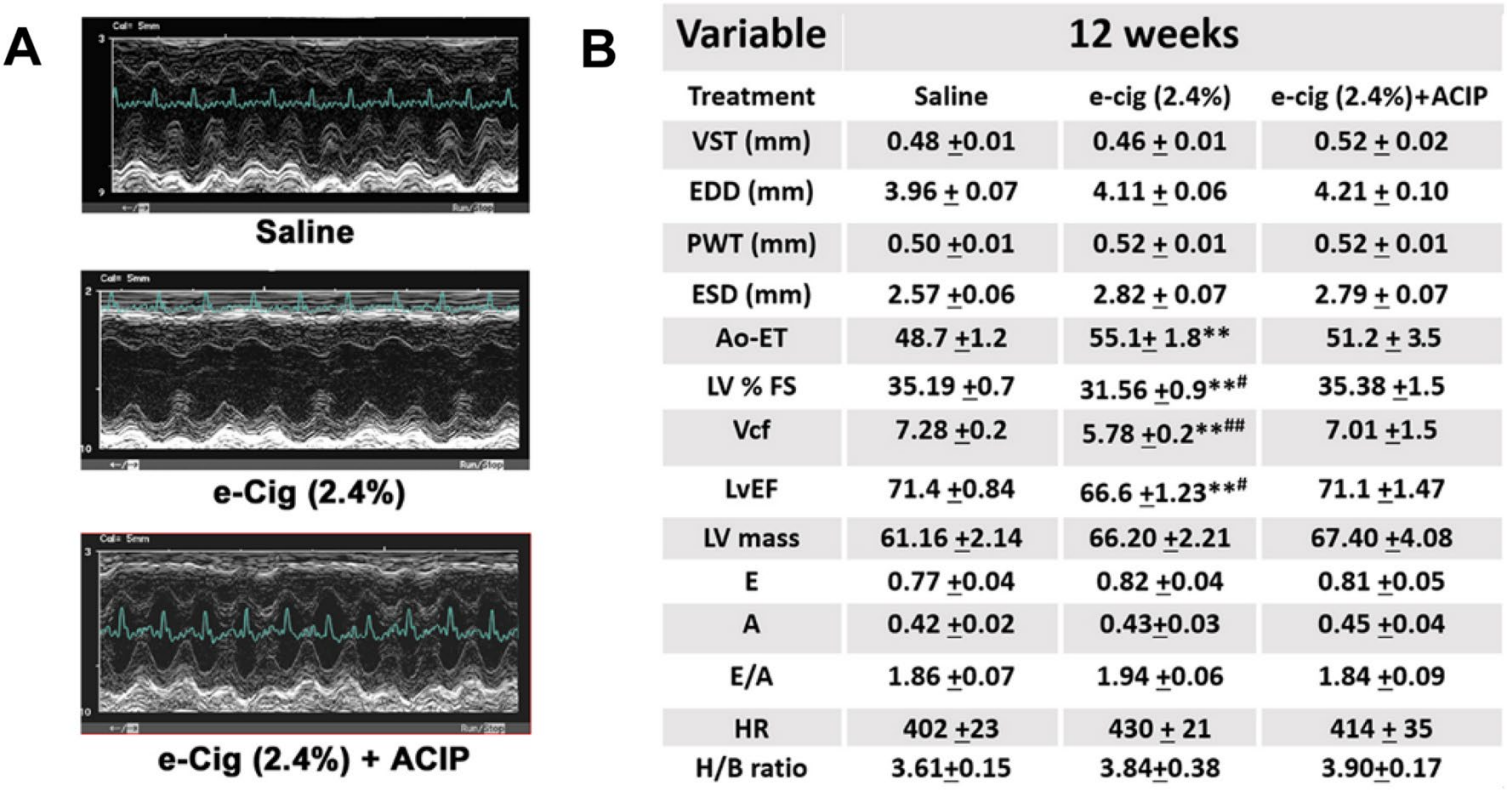
Evaluation of the cardiac function of mice treated with saline aerosol, e-cigarette (2.4% nicotine), and e-cigarette (2.4% nicotine) plus acipimox. (**A**) Representative images of M-mode echocardiogram. (**B**) Table of echocardiographic data. VST, ventricular septal thickness; EDD, end-diastolic dimension; PWT, posterior wall thickness; ESD, end systolic dimension; Ao-Et, aortic ejection time; LV%FS, left ventricle fractional shortening; VcF, velocity of circumferential fiber shortening; LvEF, left ventricle ejection fraction; LV Mass, left ventricle mass; E, early diastole (LV filling); A, atrial systole; HR, heart rate; H/B ratio, heart body ratio. Saline N = 19; e-cigarette (2.4%) N = 15; e-cigarette (2.4%) + ACIP N = 10. (Saline aerosol vs. e-cigarette (2.4%),*P < 0.05, **P < 0.01; e-cigarette (2.4%) vs. e-cigarette (2.4%) + ACIP, #*P* < 0.05 ##*P* < 0.01).

To study the physiological and morphological effects of acipimox on e-cigarette-treated mice, we performed echocardiographic measures. Figure 1A displays representative images of the M-mode echocardiogram. M-mode analysis of LV dimensions showed no changes among saline, e-cigarette (2.4%), and e-cigarette (2.4%) + ACIP (Fig. 1B). We did not observe heart/body ratio changes after 12-week treatment between the three groups (Fig. 1B). Figure 1B displays the effects of these three treatments on LV%FS, LV ejection fraction (LVEF), and velocity of circumferential fiber shortening (VcF). LV%FS, LVEF, and VcF were all reduced in mice exposed to e-cigarette (2.4%) compared with those in saline and e-cigarette (2.4%) + ACIP groups. Aortic ejection time (Ao-ET) lengthened in the e-cigarette (2.4%) group compared to the saline group. We did not detect significant changes in the left ventricular diastolic function parameters, for example, peak early diastolic (E), atrial filling velocity (A), and E/A ratio. The heart rate (HR) was alike between each group. Then, at 12 wk, mice treated with e-cigarette (2.4%) developed a diminished ventricular systolic function without change in diastolic function, and this systolic dysfunction was rescued by acipimox.

### Acipimox effects on serum lipid profile

Conventional smoking and e-cigarette vaping are associated with detrimental changes in HDL, triglycerides, and FFAs^24^. Table 1 shows that serum cholesterol, HDL, and LDL levels did not significantly change in e-cigarette (2.4%) and e-cigarette (2.4%) + ACIP groups in comparison with saline. Triglycerides were increased by e-cigarette (2.4%) (*P* < 0.01). The e-cigarette (2.4%) + ACIP group does not have a statistically significant difference from saline and e-cigarette (2.4%) groups for triglycerides. High levels of serum FFAs correlate with an increased risk for cardiac dysfunction^5^. Table 1 shows that mice exposed to e-cigarettes (2.4%) had increased levels of serum FFAs in comparison with saline (*P* < 0.01) and e-cigarette (2.4%) + ACIP (*P* < 0.05). Serum FFAs levels of mice exposed to saline and e-cigarette (2.4%) + ACIP were similar. Therefore, levels of FFAs were strongly associated with e-cigarette-induced cardiac dysfunction. Additionally, since white adipose tissue control serum FFAs via lipolysis, we measured FFAs levels within the epididymal adipose fat pads. Supplement Fig. S2 shows that e-cigarettes increased the levels of FFAs in adipose tissue and this increase was reduced by acipimox.

**Table 1:** Serum lipid profile of mice exposed to saline, e-cigarette (2.4%), and e-cigarette (2.4%) plus acipimox.

	Saline	e-cig (2.4%)	e-cig (2.4%) + ACIP
Cholesterol	184 ± 10	200 ± 26	190 ± 39
Triglycerides	78 ± 5	147 ± 18**	130 ± 17
HDL	74 ± 4	71 ± 3	78 ± 10
LDL	16 ± 2	26 ± 10	29 ± 12
FFAs	0.65 ± 0.08	1.1 ± 0.06**^#^	0.75 ± 0.06

*HDL* high-density lipoprotein cholesterol, *LDL* low-density lipoprotein cholesterol, *FFAs* free fatty acids. (N = 5 per group, saline aerosol versus e-cigarette (2.4%) **P < 0.01; e-cigarette (2.4%) versus e-cigarette (2.4%) + ACIP, ^#^*P* < 0.05). All values are means ± SEM.

### Acipimox effects on inflammatory levels of serum cytokines induced by e-cigarettes

**Figure 2:**
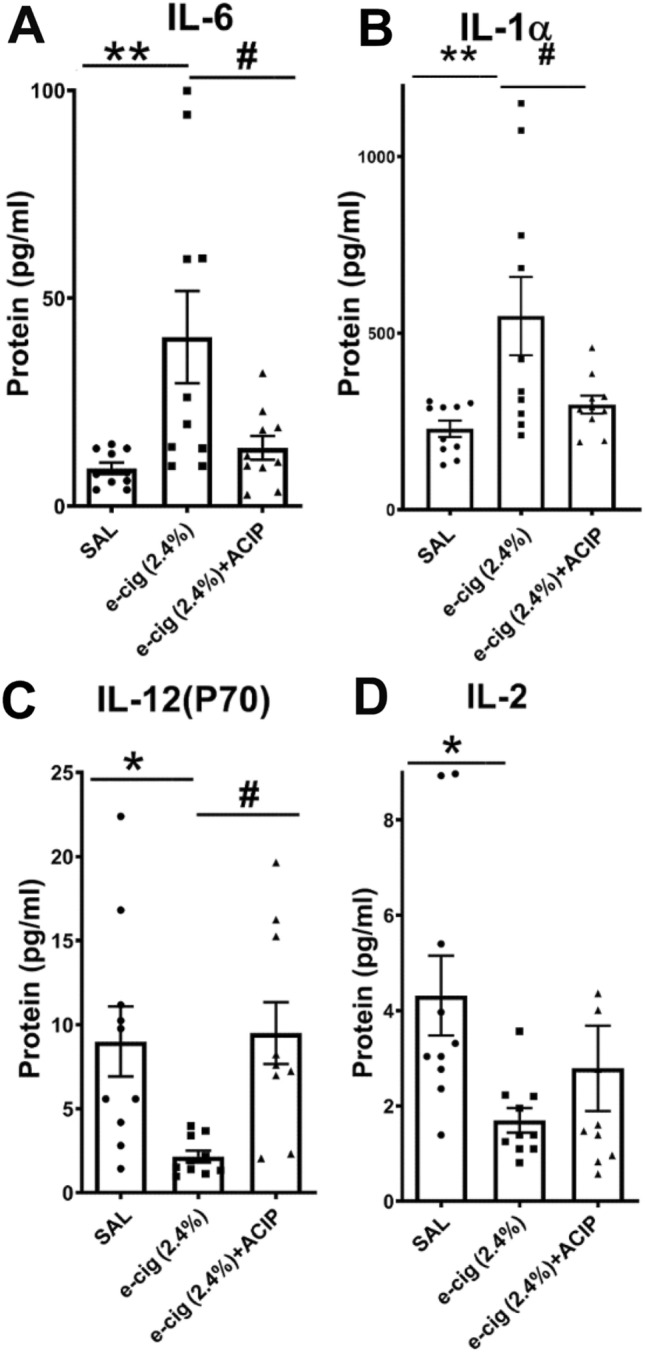
Effects of e-cigarette and acipimox on cytokines. (**A**) Interleukin 6 (**B**) Interleukin 1α (**C**) Interleukin 12(P70) (**D**) Interleukin 2. (N = 10 per group; Saline vs. e-cigarette (2.4%), *P < 0.05, **P < 0.01; e-cigarette (2.4%) vs. e-cigarette (2.4%) + acipimox, ^#^*P* < 0.05).

Numerous studies have shown that increased FFAs activate inflammatory cytokines exerting important effects on cardiovascular disease^8^. Therefore, we used a mouse cytokine Multiplex Assay to determine changes in blood serum inflammatory cytokines induced by e-cigarettes and rescued by acipimox. In Fig. 2, we showed the serum cytokines that significatively change with the acipimox treatment. For a full report of the cytokine Multiplex Assay, see Supplementary Table S1. Figure 2A shows that e-cigarette-induced an increase in levels of IL-6 (*P* < 0.01), which are rescued by Acipimox (*P* < 0.05). Figure 2B shows that e-cigarettes produced an increase in serum IL-1α (*P* < 0.01). The increased levels of IL-1α were normalized in the e-cigarette (2.4%) + ACIP group (*P* < 0.05). Interestedly, IL-6 and IL-1α are associated with inflammatory response and oxidative stress^25,26^. Figure 2C shows that serum IL-12(p70) was decreased in e-cigarette (2.4%) group in comparison with saline (*P* < 0.05) and e-cigarette (2.4) + ACIP groups (*P* < 0.05). Accordingly, cigarette smoke-induced oxidative stress suppressed the expression of IL-12(p70)^10^. Figure 2D shows that e-cigarette (2.4%) reduced IL-2, and acipimox did not affect this change. Interestedly, nicotine inhibits the production of IL-2 form mononuclear cells^9^.

### Transcriptome analysis of acipimox-mediated rescue of the e-cigarette-induced cardiac dysfunction in mice

**Figure 3:**
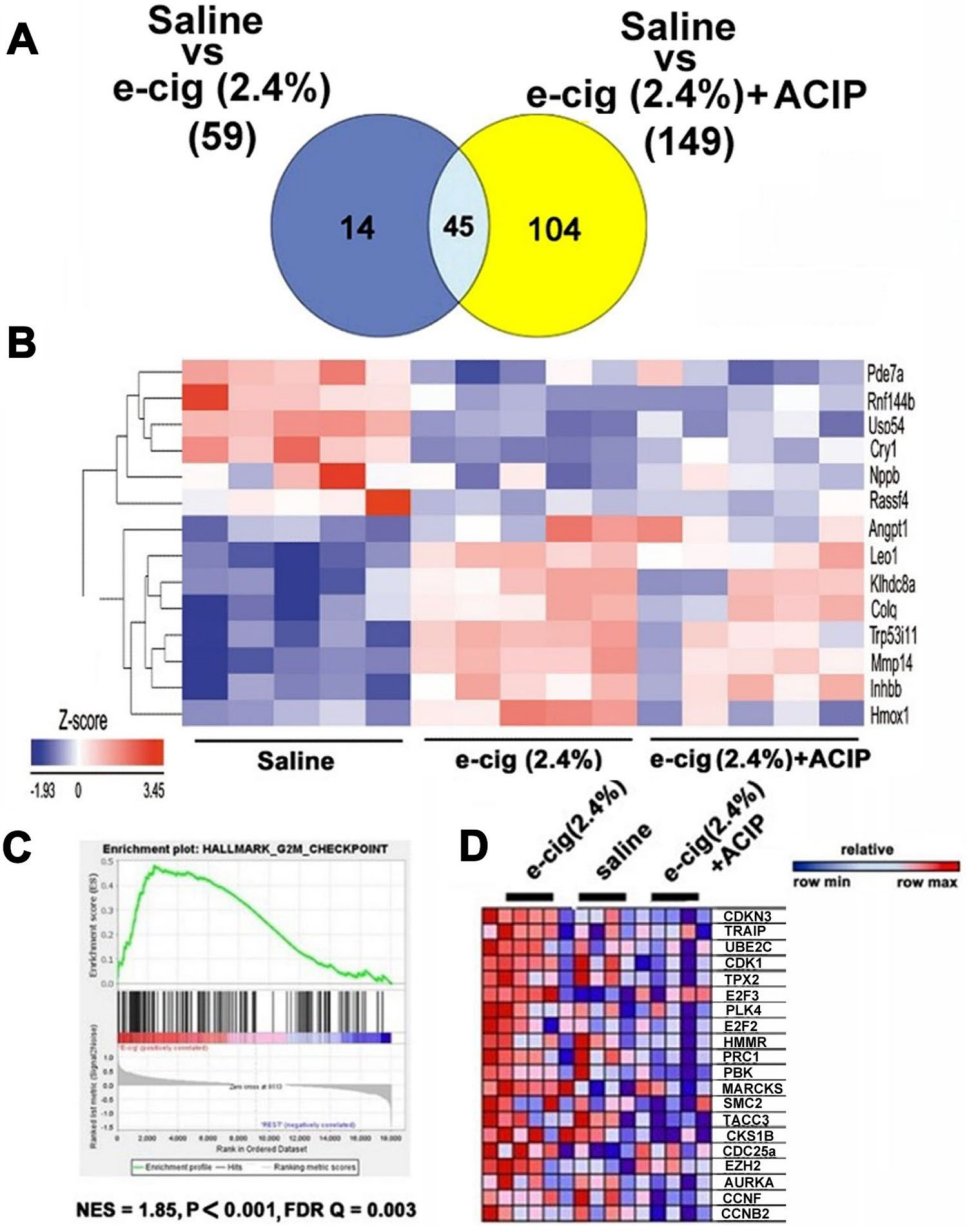
(**A**). Venn diagram representing data summary of differentially expressed genes in saline versus e-cigarette (2.4%) and saline versus e-cigarette + acipimox. (**B**) Heatmap of the genes contributing to the recue of e-cigarette induced cardiac dysfunction by acipimox. (**C**) GSEA enrichment plot presenting e-cigarette (2.4%)-induced dysregulation of genes involved with the G2/M DNA damage checkpoint as compared with saline and e-cigarette (2.4%) + ACIP. (**D**) Heatmaps of the top 20 genes from G2/M DNA damage checkpoint, as inferred from GSEA. NES = normalized enrichment score; P = nominal *P* value; FDR Q = false discovery rate Q value.

To identify the molecular pathways that mediate the effects of acipimox on e-cigarette-induced cardiac dysfunction, we used RNA-Seq analysis to profile the hearts of mice treated with saline, e-cigarette (2.4%), and e-cigarette (2.4%) + ACIP. We identified the differentially expressed genes (DEGs) of saline versus e-cigarette (2.4%). We observed 59 DEGs composed of 21 upregulated and 38 downregulated genes. The heat maps of the groups were analyzed by clustering. Probe set signal assessments were standardized to the mean throughout the mice, and the relative reading of gene expression is represented from the maximum downregulated (green) to the maximum upregulated (red). Supplement Fig. S3A displays the 2-dimensional hierarchical clustering of saline versus e-cigarette (2.4%). For saline versus e-cigarette (2.4%) + ACIP, we observed 149 altered transcripts. The 149 DEGs were composed of 23 upregulated and 126 down-regulated genes. Supplementary Fig. S3B shows the two-dimensional hierarchical clustering of saline versus e-cigarette (2.4%) + ACIP. The acipimox-rescued DEGs were defined as DEGs between saline versus e-cigarette (2.4%), but not statistically different between saline versus e-cigarette (2.4%). The Venn diagram (Fig. 3A) depicts the identification of the 14 genes that were DEGs between saline versus e-cigarette (2.4%) but were not differentially expressed between saline versus e-cigarette (2.4%) + ACIP. Figure 3B shows heat maps of the 14 genes associated with the functional rescue of acipimox. The fold changes (FC) in gene expression were reported in Supplementary Table S2. The IPA software analysis of saline and e-cigarette (2.4%) + ACIP (normal cardiac phenotype) versus e-cigarette (2.4%) shows differential expression of circadian rhythms, ferroptosis, adipogenesis pathways, G2/M DNA damage checkpoint signaling, and atherosclerosis (Supplementary Table S3).

For the threshold-free method^27^, GSEA analysis exposed an enrichment of G2/M DNA damage checkpoint in the e-cigarette (2.4%) compared with saline and e-cigarette (2.4%) + ACIP groups. Figure 3C shows the enrichment plot for G2/M DNA damage checkpoint (NES = normalized enrichment score) = 1.85; *P* value < 0.01; false discovery rate [FDR] < 0.15). Figure 3D shows the 20 top upregulated genes in the enrichment plot in Fig. 3D. For instance, TRAIP is an e-cigarette (2.4%)-upregulated gene that is ameliorated by acipimox. TRAIP is not only necessary for cell cycle progression, but it also promotes the DNA damage response following DNA replication^28^. Similarly, the mRNA of TPX2 (see Fig. 3D), which is involved in the early stages of the DNA damage response, increased in e-cigarette (2.4%) but was rescued in the e-cigarette (2.4%) + ACIP group. Therefore, e-cigarette (2.4%) induces transcriptional changes associated with the activation of DNA damage response in the heart, and this response is absent in mice exposed to e-cigarette (2.4%) + ACIP.

### Validation of RNA-seq data by qPCR

**Figure 4:**
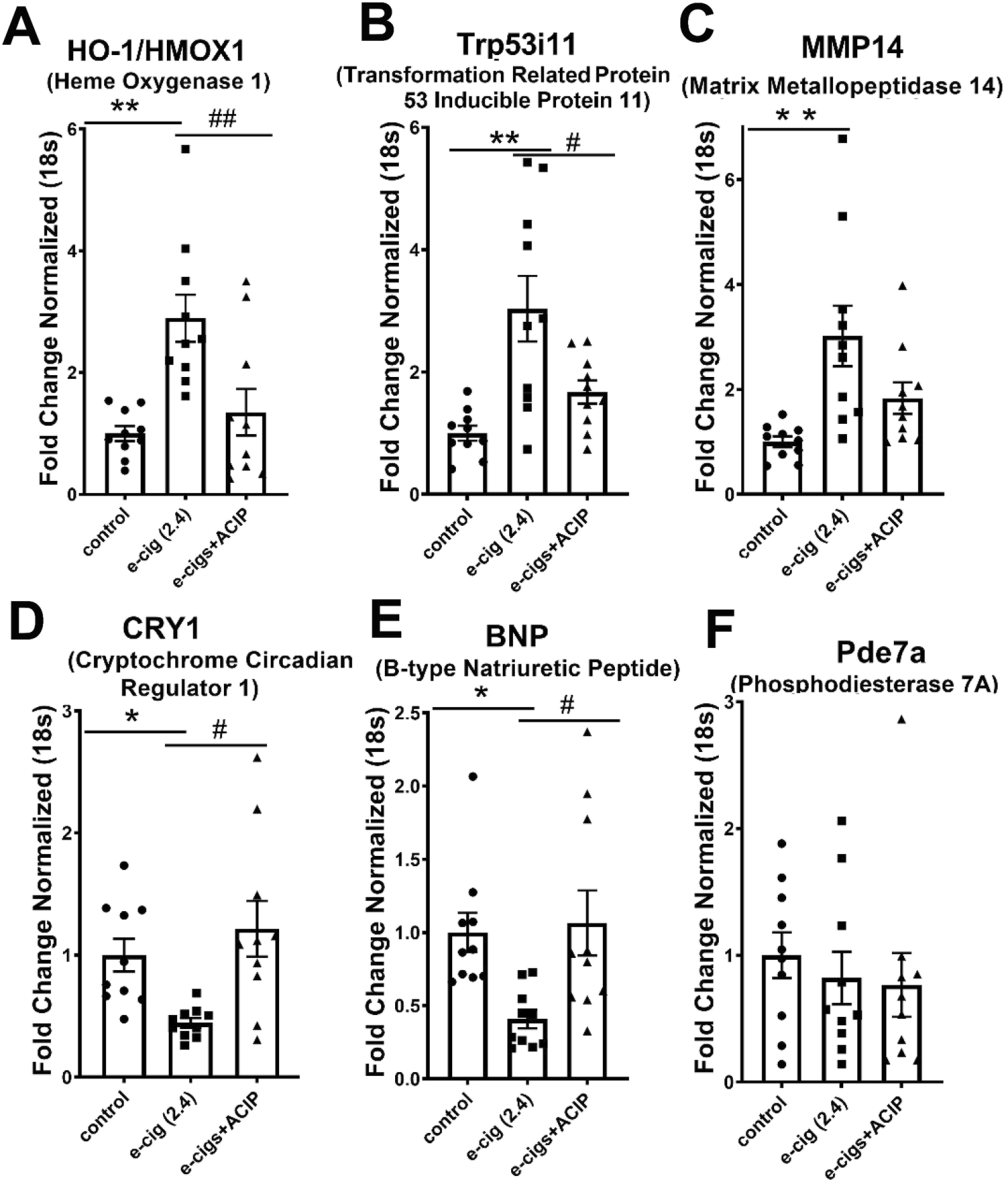
Validation of relative expression of genes obtained from RNA-seq by qPCR. Relative quantification normalized to 18 s. (**A**) HO-1, Heme oxygenase 1. (**B**) Trp53i11, Transformation related protein 53 inducible protein 11. (**C**) Mmp14, Matrix metallopeptidase 14. (**D**) CRY1, Cryptochrome circadian regulator 1. (**E**) BNP, B-type Natriuretic Peptide. (**F**) Pde7a, Phosphodiesterase 7A. (N = 10 per group, *P < 0.05, **P < 0.01).

We performed qPCR analysis of DEGs that were rescued by acipimox (Fig. 3B and Supplementary Table S2). Among the 14 genes rescued by acipimox, we identified Heme oxygenase 1 (*HO-1*), a commonly used marker of oxidative stress^29^. HO-1 is part of the response of the cells to oxidative stress and plays a role in cytoprotection against oxidative stress^30^. Figure 4 shows that e-cigarette (2.4%) increased the expression of HO-1 mRNA, and this increase was normalized in e-cigarette (2.4%) + ACIP. Tumor protein p53-inducible protein 11 (Trp53i11), one of the direct transcriptional targets of p53, is upregulated in apoptosis induced by multiple DNA damage agents^31^. Figure 4 shows that e-cigarette (2.4%) increased Trp53i11 mRNA expression in saline (*P* < 0.01) and e-cigarette (2.4%) + ACIP(*P* < 0.05). Another relevant gene rescued was Matrix Metallopeptidase 14 (*MMP14*), which is associated with cardiac fibrosis and is increased by angiotensin II^32^. E-cigarette (2.4%) increased *MMP14* mRNA expression in comparison with saline (*P* < 0.01). The changes in cryptochrome Circadian Regulator 1 (*CRY1*), a circadian factor that connects DNA damage response with circadian clock^33^, were rescued by acipimox. Figure 4 shows that e-cigarette (2.4%) reduced the *CRY1* expression in comparison with saline (*P* < 0.05) and e-cigarette (2.4%) (*P* < 0.05). B-type Natriuretic Peptide (BNP) is a marker for the diagnosis and prognosis of various cardiovascular diseases and is decreased in the obese population^34^. Cardiac BNP mRNA in treated e-cigarette (2.4%) was normalized by acipimox in comparison with saline (*P* < 0.05) and e-cigarette (2.4%) + ACIP (*P* < 0.05). *Pde7a* hydrolyzes the cyclic nucleotide second messenger with a role in autoimmune diseases^35^. We did not observe significant changes in *Pde7a* mRNA in the three groups.

### Acipimox normalizes oxidative stress induced by e-cigarettes

**Figure 5:**
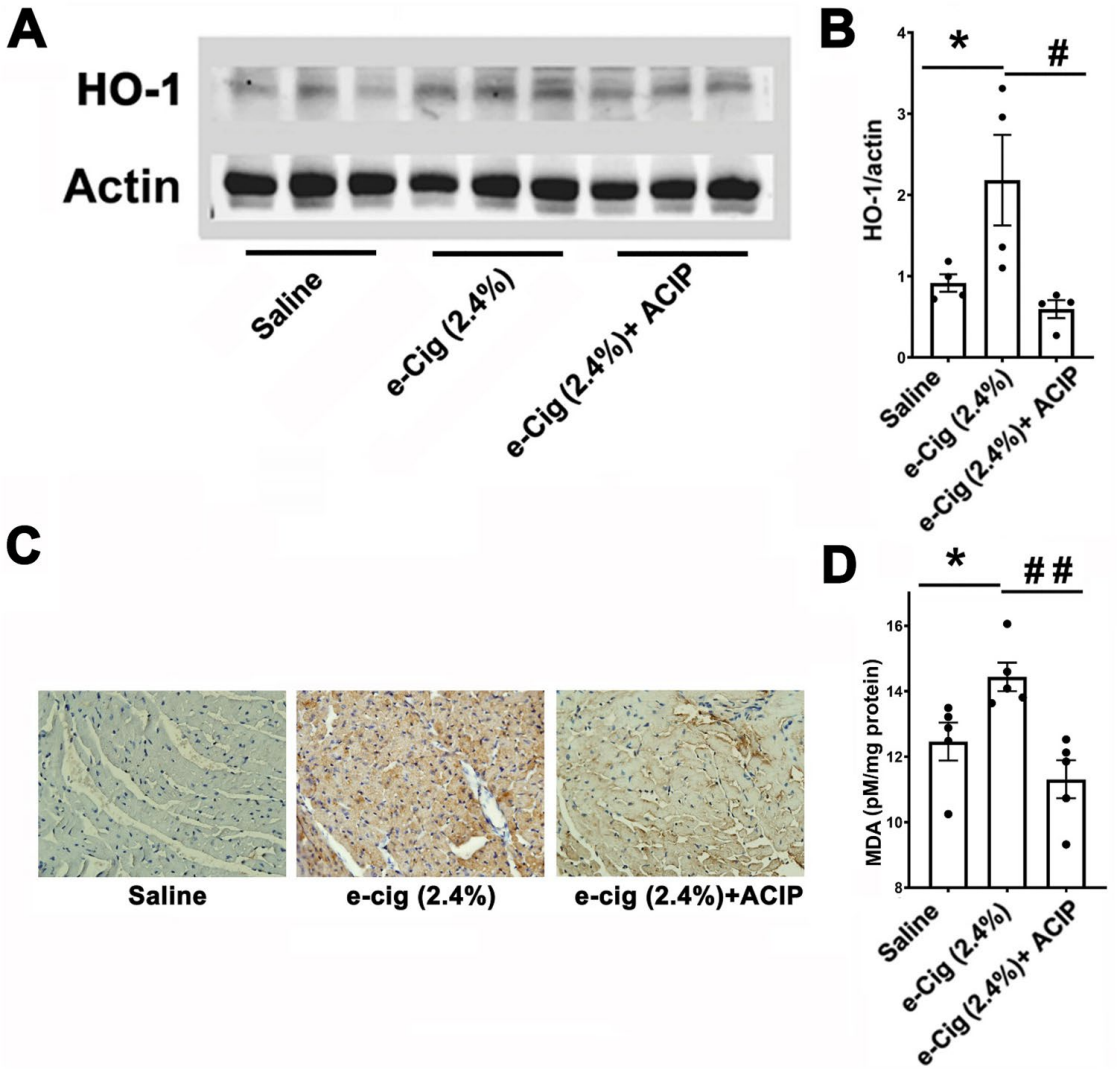
Acipimox rescues the e-cigarette induced oxidative stress. (**A**) Western blot of heart tissue showing HO-1 levels. β-actin was included as a loading control. (**B**) quantification of Western blots of HO-1 (**C**). Immunohistochemistry of ventricular sections shows increased expression of 4-HNE in e-cigarette (2.4%) compared with saline or e-cigarette (2.4%) + ACIP. (**D**) Levels of malondialdehyde (MDA). (N = 4 per group, e-cigarette (2.4%) vs. e-cigarette (2.4%) + ACIP, ^#^*P* < 0.05). All values are means ± SEM.

We have identified HO-1, a marker of oxidative stress among the 14 genes rescued by acipimox. The normalization of increased expression of HO-1 was validated by qPCR. Therefore, we determined whether these changes in mRNA levels led to increased protein levels. Western blot analysis shows that HO-1 levels were increased in e-cigarettes (2.4%)-treated hearts (Fig. 5A, B). Acipimox normalized the increased protein levels of HO-1 induced by e-cigarettes. Lipid peroxidation and the consequent generation of highly electrophilic aldehydes, such as 4-hydroxynonenal (4-HNE) are used as a marker of oxidative stress. The increased levels of HO-1 were accompanied by an increase in the immunostaining of 4-HNE in left ventricular sections, which was normalized in the e-cigarette (2.4%) + ACIP group (Fig. 5C). Malondialdehyde (MDA) is used as a marker of a lipid peroxidation and oxidative stress. Figure 5D shows that e-cig (2.4%) increased MDA in cardiac tissue. This increase in MDA was normalized by acipimox. Overall, Fig. 5 shows that oxidative stress was exacerbated by e-cigarette (2.4%) and normalized by inhibition of lipolysis with acipimox.

### E-cigarette (2.4%)-induced DNA damage was normalized by acipimox

**Figure 6:**
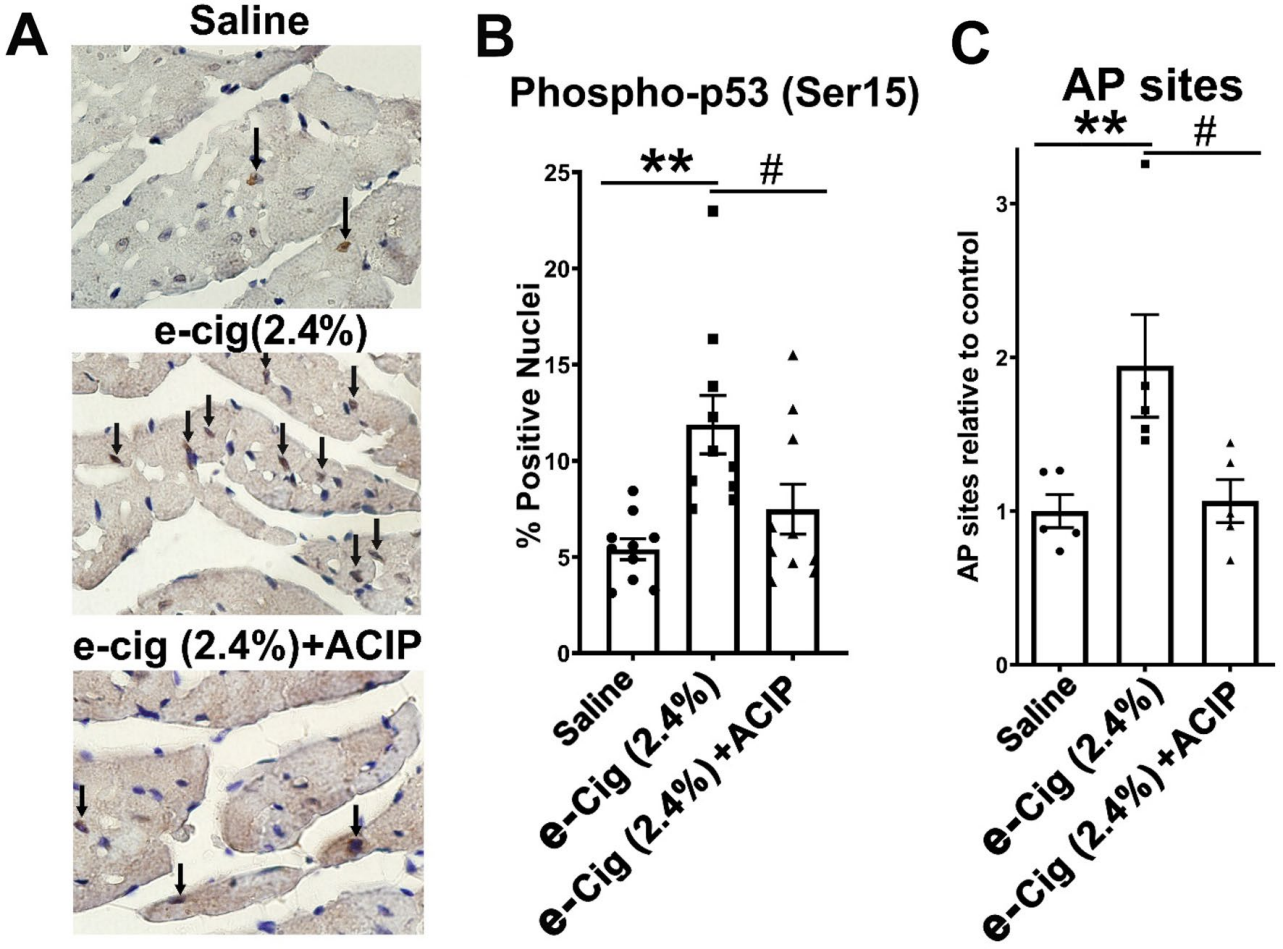
E-cigarette-induced DNA damage is inhibited by acipimox. (**A**) Representative heart sections from mice treated with saline, e-cigarette (2.4%), and e-cigarette (2.4%) + ACIP. (**B**) Quantification of the data showing that e-cigarette (2.4%) exposure increases p-P53-positive nuclei, and acipimox reduces the e-cigarette (2.4%) induced p-P53 increase. (**C**) E-cigarette (2.4%) exposure increases AP sites, and acipimox blunt the e-cigarettes induced AP sites increase. P-P53, N = 10 per group. AP sites, N = 5, per group, *: *P* < 0.05, **: *P* < 0.01, saline aerosol versus e-cigarette (2.4%). #: *P* < 0.05, e-cigarette (2.4%) versus e-cig (2.4%) + ACIP.

The unbiased analysis of the RNA-seq data by GSEA and IPA analysis shows a phenotype associated with DNA damage that is rescued by acipimox. Since TRP53i11 is a direct target of P53, we study the phosphorylation of P53 in ser15 (p-P53), a marker of DNA damage^16^. To determine the effects of chronically increased lipolysis on P53 pathway activation, we performed immunohistochemistry of p-P53. Figure 6A shows representative ventricular sections of mice with different treatments stained to determine the fraction of cells with nuclear-localized p-P53. The black arrows in Fig. 6A show the nuclear localization of p-P53 staining. Figure 6B shows the nuclear-localized p-P53 calculated from examining 1000 nuclei for each animal and graphed. Therefore, mice exposed to e-cigarettes had increased p-P53 staining, which was normalized by acipimox. We showed that acipimox reduced oxidative stress induced by e-cigarettes. Apurinic/apyrimidinic (AP) sites are a marker of oxidative DNA damage. Therefore, after extracting cardiac genomic DNA, we studied the nuclear contents of AP site lesions. Figure 6C shows that AP site lesions were increased in mice exposed to e-cigarette (2.4%). In contrast, in e-cigarette (2.4%) + ACIP, the levels of AP site lesions were not significantly changed compared with saline. These data suggest that the effects of e-cigarettes on cardiac DNA base damage were due to increased level of ROS in a lipolysis-dependent manner.

### Acipimox rescued e-cigarette (2.4% nicotine)-induced cardiac ultrastructural abnormalities

**Figure 7:**
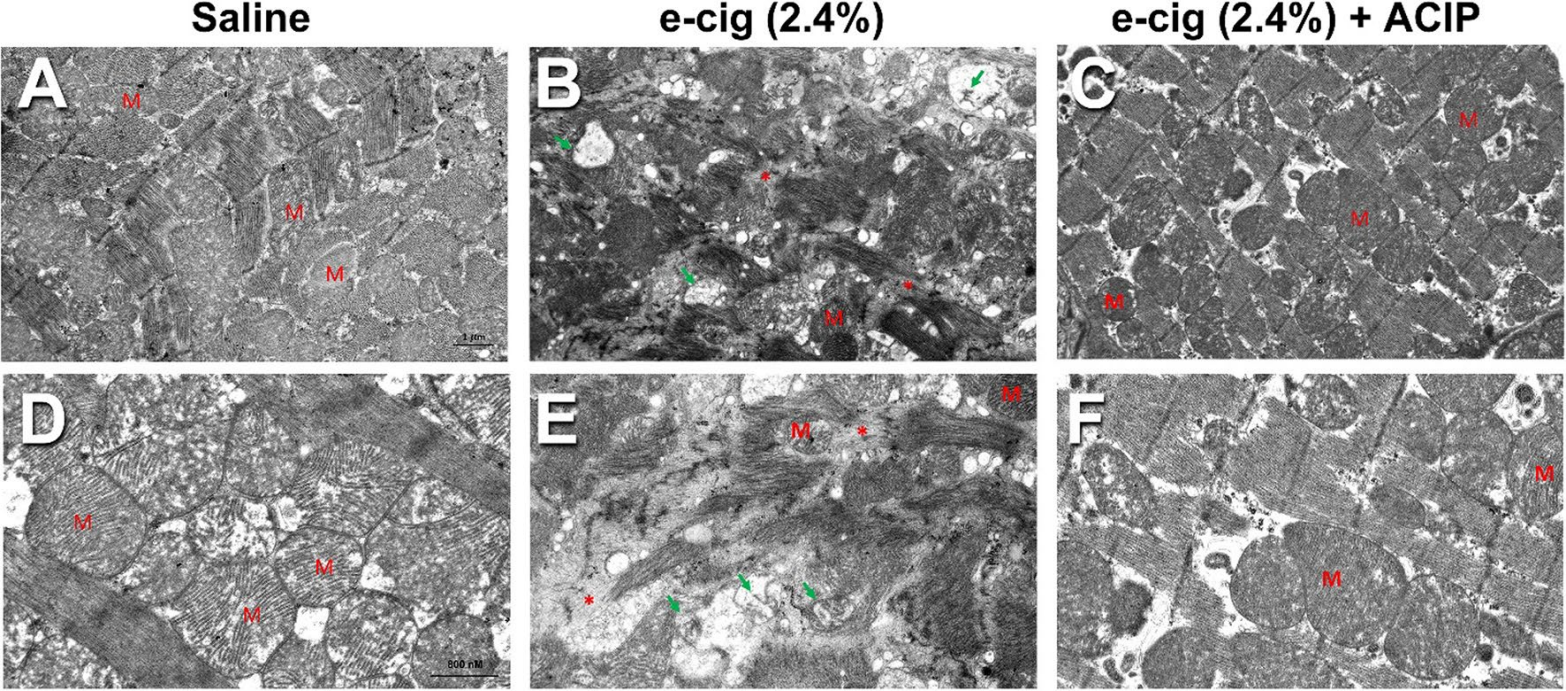
The E-cigarette induced ultrastructural abnormalities are normalized by acipimox. Representative TEM images of left ventricular myofibrillar architecture in various treatment groups (**A**–**C**, lower magnification. Scale bar = 1 μm) (**D**–**F**, higher magnification. Scale bar = 800 nm). CMs from saline- (**A** and **D**) exposed mice show normal myofibrillar architecture and sarcomeric pattern with abundant mitochondria (M). In contrast, CMs from e-cigarette (2.4%)-exposed (**B** and **E**) show varying degrees of abnormalities indicative of cardiomyopathy, including myofbrillar thinning, derangement, destruction (asterisk), mitochondrial vacuolization with crystolysis (arrow) These abnormalities were rescued in mice exposed to e-cigarette (2.4%) plus acipimox (**C** and **F**).

We then tested if the e-cigarette (2.4%)-induced cardiac structural changes described in our prior publication^4^ were normalized by acipimox. We used transmission electron microscopy (TEM) to assess left ventricular myofibrillar composition in the experimental groups. Cardiomyocytes (CMs) from saline-treated mice showed normal myofibrillar architecture and sarcomeric pattern with normal mitochondria shown in Fig. 7A, D. In contrast, CMs treated with e-cigarette (2.4%)-exposed mice exhibited vacuolated mitochondria with crystolysis and myofbrillar thinning, derangement, and destruction (Fig. 7B, E). However, e-cigarette (2.4%) + ACIP mice-treated hearts showed normalization of mitochondrial ultrastructure as well as myofibrillar architecture and sarcomeric pattern (Fig. 7C, F). Therefore, acipimox normalized cardiac ultrastructural abnormalities associated with e-cigarette-induced cardiac dysfunction.

## Discussion

Obese adolescents have a higher rate of e-cigarette use^3,23^. In mice fed with a NCD, nicotine produces a modest impact on the cardiovascular system. For example, C57BL/6J mice on NCD have been exposed to e-cigarettes for 60 weeks^36^ without showing a decrease in LV%FS, but with impaired vasodilation^36^. Therefore, to study the cardiac effects of e-cigarettes, we used a relevant model with a reliable phenotype, wild-type mouse model on HFD^19^. In the cardiovascular system, the synergistic effect of HFD and nicotine have been studied by our and other groups^1,37^. Using the HFD mice model to study synergistic factors that increase morbidity or mortality in obese people who vape has relevance to the vaping company's young target population and the health of the general population. We have previously established a protocol for the induction of cardiac dysfunction by e-cigarettes in a nicotine-dependent manner^4,19,38^.

Acipimox is a derivative of Nicotinic acid (Niacin). The receptor for this family has been named in several ways HCA2/ GPR109A/ HM74/ PUMA-G^18^. Humans have two isoforms of niacin receptor, whereas rodents have only one^18^. The nicotinic acid-induced decrease in FFAs is reduced in mice lacking HCA2/PUMA-G. Therefore, the effect on FFAs of the niacin compound family is mediated through the HCA2/PUMA-G^39^. Several groups have shown that HCA2 is localized mainly in macrophages and adipose tissue^39–41^, and the molecular details of the acipimox binding to this receptor are well established^17,40^. In the current study, mice were exposed to e-cigarette aerosol in a chronic intermittent protocol with 2.4% nicotine for 12 weeks. In this model, we have shown nicotine and cotinine levels similar to clinically relevant concentrations found in e-cigarette users and smokers^19^. At 12 weeks of e-cigarette (2.4%) treatment, echocardiography showed a marked decrease in systolic function without changes in gross cardiac morphologic parameters. The treatment with acipimox rescued the parameters associated with systolic dysfunction (LV%FS, LvEF, and VcF) showing that the inhibition of lipolysis normalized systolic dysfunction induced by e-cigarettes. E-cigarette (2.4%)-treated mice displayed prolonged Ao-ET, and acipimox showed a tendency to normalize Ao-ET. However, we did not observe a full rescue of Ao-ET by acipimox. Therefore, a small component of e-cigarette effects on Ao-ET may be lipolysis-independent. Overall, these data suggest that acipimox protects most of the cardiac function from the detrimental effects of e-cigarettes.

FFAs have been broadly studied for their contribution to the induction of metabolic changes that lead to metabolic syndrome and adverse cardiovascular outcomes^5^. Inhibition of lipolysis using acipimox reduced nicotine plus HFD-induced hepatic steatosis^42^. In this model, we have shown that nicotine is necessary to produce the e-cigarette-induced FFAs increase^19^. In Table 1, we showed that e-cigarettes produce an increase in FFAs and triglycerides, and acipimox fully normalized this increase in FFAs. Likewise, we observe an increase of FFAs in epididymal adipose fat pads, which was reduced by acipimox (Supplement Fig. S2).

The systemic activation of nAChRs by nicotine produces a complex mix of anti-inflammatory and proinflammatory effects. Nicotine binding to nAChRs led to a release of adrenaline from the adrenal medulla and noradrenaline from postganglionic sympatric nerves, activating the sympathetic nervous system^1^. Interleukin-6 (IL-6) signaling mediates inflammation leading to cardiovascular complications. IL-6 is independent predictor of Type 2 diabetes and associated cardiovascular events^43^. E-cigarettes increase the release of IL-6 from fibroblasts, macrophages, and epithelial cells^25^. Additionally, the FFA palmitate increases expression of IL-6 in human coronary artery endothelial cells^44^.

Figure 2A shows that the reduction of FFAs with acipimox led to normalizing IL-6 levels. The proinflammatory cytokine IL-1α is present in virtually all cell types and released by dying cells, including CMs^45^. Additionally, IL-1α is reported to increase upon e-cigarette exposure^25^. Figure 2B shows that increased levels of IL-1α induced by e-cigarettes were normalized by acipimox. IL-12p70 protein production was suppressed by cigarette smoke extract in a nicotine-dependent manner, and this reduction was normalized by anti-oxidants^10^. NF-E2–related factor 2 (Nrf2), an oxidative stress sensor, represses the expression of IL-12p70. We observed that e-cigarettes not only repressed IL-12p70 expression (Fig. 2C), but also increased oxidative stress. Therefore, since acipimox normalized oxidative stress generated by e-cigarette exposure, the normalization of IL-12p70 by acipimox may be produced by this reduction of oxidative stress.

IL-2 signaling promotes the development and homeostasis of regulatory T cells, a subpopulation of lymphocytes. Regulatory T cells inhibit excessive and uncontrolled immune responses. Therefore, in clinical trials, IL-2 has been used to treat several cardiovascular conditions, including cardiac remodeling after myocardial infarction^46^ and vascular inflammation^47^. Nicotine and cigarette smoke extracts inhibit the production of IL-2 in human mononuclear cells^9^. Figure 2D shows that e-cigarettes reduced the expression of IL-2. However, we did not observe a significant difference between e-cigarette (2.4%) and e-cigarette (2.4%) + ACIP. Therefore, the inhibitory effect of e-cigarette (2.4%) on IL-2 was, at least in part, independent of nicotine-induced lipolysis.

This study was designed to identify early cardiac transcriptomic changes during the induction of e-cigarette-induced cardiac dysfunction, which were rescued by inhibition of lipolysis. Therefore, mice were euthanized immediately after the early detection of systolic dysfunction to identify the mechanisms underlying the onset of e-cigarette (2.4%)-induced cardiac dysfunction. The differential gene expression analysis of the e-cigarette (2.4%) treatment led to the dysregulation 59 genes in comparison with saline (Fig. 3A). Additionally, e-cigarette (2.4%) + ACIP treatment led to the dysregulation of 149 genes in comparison with saline. Further analysis revealed that 14 genes were changed by e-cigarette (2.4%) and rescued by the acipimox treatment. Nevertheless, despite its normalizing effects, acipimox induced the change of 104 genes that were not altered by e-cigarette (2.4%) treatment. Therefore, we cannot exclude an unnoticed physiological change by acipimox independent of the e-cigarette functional rescue.

Among the rescued genes, we observed increases in HO-1, TRP53i11, and MMP14. HO-1, an Nrf2-regulated gene, is part of the cellular response to oxidative stress and is commonly used as a marker of ROS^29^. We observed that HO-1 was increased by e-cigarettes and normalized by acipimox. Together with the e-cig (2.4%)-induced increase of MDA levels, which were reduced by acipimox, these finding suggests that the rescue of oxidative stress by acipimox reversed the e-cigarette-induced phenotype. TRP53i11/Tp53i11 is a direct p53 target gene. P53 is activated by phosphorylation, leading to the transcription of genes critical for the cellular response to DNA damage. Consistent with the differential gene expression analysis, GSEA and IPA analysis showed an increased G2/M DNA damage checkpoint in the e-cigarette (2.4%)-treated heart compared with saline and e-cigarette (2.4%) + ACIP. The heart is composed of 30–35% of CMs, with the remaining 65–70% being composed mostly of non-muscular cells that undergo mitosis^48^. Therefore, a DNA damage stimulus, which exerts its effects on cardiomyocyte and non-cardiomyocyte cells, may produce an effect on the G2/M DNA damage checkpoint. In the development of cardiac remodeling associated with fibrosis, the matrix metalloproteinase, MMP14, stimulates cardiac fibroblast migration^32^. These changes have a profound effect on cardiac physiology. Consistent with the echocardiographic changes, we have shown that e-cigarette (2.4%) increased MMP14 mRNA, which was normalized by acipimox. In contrast, among the rescued genes in the RNA-seq analysis, we observed a decrease in CRY1 and BNP genes. Circadian genes control not only circadian rhythms but also control inflammatory pathways. CRY1 is postulated as a mediator of the cross-talk between the circadian clock and the immune system. CRY1 can reduce inflammation through inhibition of TNF-α transcriptional activation^49^. Additionally, inflammatory response of patients with an increase in IL-6 was associated with a decrease in CRY1 mRNA. Accordingly, we have found that e-cigarette (2.4%) decreased CRY1 mRNA, and this decrease was normalized by acipimox. Overall, of the 6 genes tested, acipimox-normalized 4 genes tested with qPCR. Although we established a correlation between specific genes with the rescue of e-cigarette-induced cardiac dysfunction, their precise role in phenotype induction will need further investigation.

The BNP levels and body mass index are inversely correlated^50^. In humans, higher circulating levels of BNP are associated with greater insulin sensitivity, in addition to their association with heart failure^51^, especially if it is uncompensated. Accordingly, mice overexpressing BNP are protected against glucose intolerance induced by HFD via increased mitochondrial biosynthesis and fat oxidation^52^. Since we have observed that e-cigarettes induced cardiac dysfunction through mechanisms shared with metabolic disease, inhibiting BNP mRNA in this phenotype was not unexpected, especially as the mice in our study did not have hemodynamically unstable heart failure^51^.

FFAs can increase the mitochondrial generation of ROS. Increased ROS by FFA-induced mitochondrial dysfunction has been postulated as a major mechanism of diabetic cardiomyopathy^5^. We observed a rescue of HO-1 protein levels induced by e-cigarette (2.4%). Accordingly, e-cigarette induction of lipid peroxidation product 4-HNE, was rescued by acipimox treatment. Oxidative stress activates a wide variety of signaling kinases and transcription factors associated with cardiac dysfunction and stimulates extracellular matrix remodeling and DNA damage^5^.

The induction of DNA damage by e-cigarettes has been shown in several tissues^16^, including the heart^15^. The p53 pathway senses oxidative DNA damage and modulates base excision repair in response to persistent oxidative stress. Consistently, in the lungs of e-cigarette users, nuclear localization of p53 staining is increased^53^. GSEA analysis suggested an increase in DNA damage associated with e-cigarette treatment, and qPCR showed increased transcription of a P53 target, TRP53i11, normalized by acipimox. Therefore, we studied the DNA damage-dependent phosphorylation of P53 at Ser-15 and its nuclear localization immunostaining. We showed that e-cigarette treatment increases nuclear localization of p-P53, which is reversed by acipimox. We showed increased levels of AP sites in the hearts of mice treated with e-cigarettes, and acipimox rescued the levels of AP sites. The activation of P53 promotes apoptosis. Consistently, e-cigarettes promote apoptosis in IPS-derived cardiomyocytes^54^ and we have shown the role of apoptosis in the model used in this work^19^. Consistently, FFA-induced ROS produce DNA damage and cardiac dysfunction^5^. In hearts exposed to e-cigarettes, we observed CMs ultrastructural abnormalities indicative of cardiomyopathy and contractile dysfunction. The ultrastructural changes in CMs are structural manifestations of altered cardiac LV function in response to e-cigarette (2.4%) exposure. Acipimox normalized these ultrastructural abnormalities in direct correlation with echocardiographic data. Altogether, these observations suggest the reduction of e-cigarette-induced lipolysis by acipimox reversed ultrastructural abnormalities and oxidative stress associated with subsequent oxidative DNA damage and P53 activation.

E-cigarettes have many effects on the cardiovascular system due to their various components^1^. Nicotine has proinflammatory and anti-inflammatory effects. Also, e-cigarettes produce an inflammatory activation of neutrophils and macrophages^55,56^. Here, we have dissected the specific effect of nicotine associated with lipolysis and increased FFAs. Our results demonstrate that increased lipolysis is necessary for several adverse effects of e-cigarettes on cardiac structure and function in mice. These studies suggest that lipolysis inhibitors such as acipimox could effectively reduce the cardiovascular effects of e-cigarettes. Future studies are needed to evaluate the effects of lipolysis inhibitors on e-cigarette users in clinical settings. Further work will be needed to understand if inhibition of DNA damage or oxidative stress is sufficient to rescue the effects of e-cigarettes. The strengths of our study include using an e-cigarette delivery technology mimicking human vaping with a commercially available brand of e-cigarettes. Furthermore, the HFD mice model represents common chronic diseases among e-cigarette users^3,23^.

E-cigarettes are relatively new, and studies are needed to determine their long-term effects. This study was primarily focused on determining the role of lipolysis on the cardiac effects of e-cigarettes in the context of obesity. Since we have described mechanisms for the effect of e-cigarettes on the heart, which is shared with metabolic disease, it was plausible to foresee a robust effect in the HFD mice model. Therefore, these shared mechanisms described here call for future studies investigating the impact of e-cigarettes on cardiovascular disease in the obese human population.

## Methods

### Animals

Animal handling and experimentation were in accordance with the recommendation of the American Veterinary Medical Association and were approved by The Lundquist Institute Institutional Animal Care and Use Committee. The animal study is reported in accordance with ARRIVE guidelines (https://arriveguidelines.org). Male C57BL/6J wild type mice were purchased from the Jackson Laboratory (Bar Harbor, ME). Mice were housed 5 per cage under controlled temperature (22 °C) and photoperiod (12-h light and 12-h dark cycle). At eight weeks of age, mice were started on a HFD with 60% of calories derived from fat (D12492; Research Diets). Mice were exposed to e-cigarette aerosol from bluCig PLUS Classic tobacco E-cigarettes containing high (2.4% nicotine) (purchased on blu.com website) for 12 weeks. For controls, mice were exposed to saline aerosol (Afasci Inc, Burlingame, CA). Acipimox (0.05%) was administered via the drinking water as described^42^. After isoflurane anesthesia, mice were decapitated. Supplementary Figure S1 shows the schematic and timeline of the experimental model.

### E-cigarette aerosol generation and rodent exposure system

We described the e-cigarette aerosol generation before^4,38^. In short, the system encompasses an aerosol exposure chamber that keeps up to five free-moving mice, an activation control unit, and e-cigarette holders. The e-cigarette activation is intermittent and fresh airflow is kept. The vaping event was every 30 min. For each vaping event, E-cigarette exposure protocol: activation for 4 s is a puff; 6 puffs per vaping event with an inter-puff interval of 26 s. Mice were treated with intermittent e-cigarette aerosol (24 vaping episodes) for 12 h at night. When mice were not treated with e-cigarettes, mice were placed in their home cages without any aerosol exposure. Water and food were provided ad-lib during both dark and light phases.

### Lipid profile

Blood was collected from overnight-fasted mice at the time of euthanasia. For serum preparation, blood was allowed to clot prior to centrifugation at 10,000× *g* for 10 min on ice. Analysis of the lipid panel (6290 rodent lipid panel) was performed at the W. Sacramento IDEXX BioAnalytics. Cholesterol, triglycerides, HDL, and LDL were determined using standard clinical chemistry Laboratory from serum samples run on a Beckman Coulter AU680 automated chemistry analyzer validated for rodent serum samples. FFAs levels were quantified using the manufacturer's protocol (BioVision, Cat# K612-100).

### Echocardiography

Following the 12-week exposure period, the mice were screened by echocardiography at the Mouse Physiology Core Laboratory at UCLA Department of Physiology. Cardiac function was evaluated by non-invasive ultrasound echocardiography under light isoflurane sedation (0.5–1.0%) to prevent movement and cardio-depression. Data were acquired using a 2D-guided M-Mode and spectral Doppler imaging with a Siemens Acuson Sequoia Model C256 equipped with a 15L8 15 MHz probe (Siemens Medical Solutions, Malvern, CA). Mice were evaluated to obtain heart dimension and function measurements including LV chamber size, wall thickness, end-diastolic dimension (EDD), end-systolic dimension (ESD), LV fractional shortening (LV%FS), velocity of circumferential fiber shortening (VCF) and LV ejection fraction (LVEF)^57–59^.

### RNA-seq analysis

RNA was isolated from left ventricles (n = 5 in each group). RNA quality was assessed with an Agilent 2100 Bioanalyzer. RNA samples exhibited clear 28S and 18S rRNA peaks and demonstrated an RNA integrity number (RIN) greater than 8. Libraries for RNA-Seq were prepared with KAPA Stranded RNA-Seq Kit. The workflow consists of mRNA enrichment, cDNA generation, and end repair to generate blunt ends, A-tailing, adaptor ligation and PCR amplification. Different adaptors were used for multiplexing samples in one lane. Sequencing was performed on Illumina HiSeq 3000 for a single read 50 run. Data quality check was done on Illumina SAV. Demultiplexing was performed using the Illumina Bcl2fastq2 v 2.17 program.

We sequenced more than 15,000 genes for RNA-seq analysis. The reads were first mapped to the latest UCSC transcript set using Bowtie2 version 2.1.0^60^ and the gene expression level was estimated using RSEM v1.2.15^61^. TMM (trimmed mean of M-values) was used to normalize the gene expression. The data were analyzed in two ways to define a gene list from omics data: For the IPA analysis and identity rescued genes, we filtered the data by a threshold (*P* < 0.01 and fold-change > 1.5), and for Gene Set Enrichment Analysis (GSEA), we studied all genes based on their differential expression rank without prior gene filtering. Differentially expressed genes were identified using the edgeR program^62^. Genes showing altered expression with *P* < 0.05 and more than 1.5 fold changes were considered differentially expressed. The pathway and network analysis was performed using Ingenuity Pathway Analysis (IPA). The canonical pathways generated by IPA are the most significant for the uploaded data set.

### Quantitative PCR

Left ventricular RNA from mice treated with saline, e-cigarette (2.4%), and e-cigarette (2.4%) + ACIP was extracted with TRIzol Reagent (Invitrogen) using a Pyrex homogenizer. Purity of RNA was determined by 260/ 280 ratio using NanoDrop 2000 (Thermo Fisher Scientific) and 260/280 ratio greater than 1.9 was considered as highly purified RNA. The cDNA was prepared using the High Quality RNA to cDNA kit (Applied Biosystems). The quantitative PCR (qPCR) was done using Step-One plus RT-PCR system (Life Technology) with an SYBR Green PCR Master Mix (Applied Biosystems). All reactions were analyzed in triplicate, and 10 mice from each group were tested. Data were normalized to 18S RNA transcripts using the 2^−ΔΔCt^ method for relative quantitation of gene expression. The primers used for real-time PCR are as follows: *HO-1*: (F) 5′-GCCGAGAATGCTGAGTTCATG-3′ and (R) 5′-TGGTACAAGGAAGCCATCACC-3′; Trp53i11: (F) 5′-GGGGCTCAGGGTCTGGCAGT-3′ and (R) 5′-CCGTAGAGGCGGATGGGGGT-3′; *Mmp14*:(F) 5′-CCCTAGGCCTGGAACATTCT-3′ and (R) 5′-TTTGGGCTTATCTGGGACAG-3′;

*CRY1:*(F) 5′-CACTGGTTCCGAAAGGGACTC-3′ and (R) 5′-CTGAAGCAAAAATCGCCACCT-3′;*BNP*: (F) 5′-AAGCTGCTGGAGCTGATAAGA-3′ and (R) 5′-GTTACAGCCCAAACGACTGAC-3′; *Pde7a*: (F) 5′-GCAGAGACGTGGAGCTATTTC-3′ and (R) 5′-CTCAAATGCAGCATTGGCATC-3′; and *18s* (F) 5′-GTAACCCGTTGAACCCCATT-3′ and (R) 5′-CCATCCAATCGGTAGTAGCG-3′.

### Serum cytokine analyses

A DropArray 96-well plate (Curiox) and mouse Milliplex magnetic bead kit with a panel of 32 analytes (Millipore, Cat # MCYTMAG-70K-PX32) was utilized following manufacturer's instructions. In short, 5 μL of serum were mixed with 5 μL magnetic beads and incubated overnight at 4°C while shaking. After washing the plates with wash buffer (PBS with 0.05% Tween-20 and 0.1% BSA) in a DropArray LT Washing Station MX96, 5 μL of detection antibody was put in and incubated for 1 h at room temperature. 5 μL streptavidin–phycoerythrin conjugate was then added to the mix and incubated for another 30 min. Following 3× washes, we resuspended the beats in sheath fluid. Fluorescence was measured using a Luminex 200. A MILLIPLEX Analyst 5.1 software was used to analyze the data.

### Electron microscopy

For transmission electron microscopic studies, ventricles were dissected, and portions of the left ventricles were fixed in 2.5% glutaraldehyde in 0.05 M sodium cacodylate buffer (pH 7.4). Portions of glutaraldehyde-fixed left ventricles were further diced into small pieces, post-fixed in 1% osmium tetroxide, and embedded in Epon 812 as described previously^42,63^. Thin sections from selected tissue blocks were cut with an LKB ultramicrotome, stained with uranyl acetate and lead citrate, and examined with a Hitachi electron microscope (Hitachi, Indianapolis, IN, USA). Special emphasis were given to key structural changes associated dilated cardiomyopathy, including nuclear abnormalities, myofibrillar derangement, thinning and destruction, intramyocardial lipid accumulation, mitochondrial vacuolization and crystolysis, and mitophagy^64^.

### Immunohistochemistry

For determining lipid peroxidation, a final product of oxidative stress, the 4-HNE staining was performed. Hearts from each group were fixed with a 4% paraformaldehyde solution. Paraffin-embedded left ventricular sections were immunostained with mouse monoclonal 4-HNE antibody (1:200, Abcam Cat# ab48506). 4-HNE antibody was with biotinylated secondary antibodies (and VECTASTAIN Elite ABC HRP kits (Vector Laboratories, Cat# PK6100) and developed with DAB (Vector, SK-4100) according to the provider’s instructions.

For p53 translocation to the nucleus, Paraffin-embedded sections were incubated with an antibody against phosphorylated p53 (p-p53) as a biomarker of DNA damage. Slides were first incubated with anti-p-p53 mouse antibody (1:100, Catalog #9284, Cell Signaling Technology, Boston, MA) and then incubated with anti-rabbit (1:200, Catalog #PK-6101, Vector Laboratories, Newark, CA) antibody, followed by application of DAB substrate (Catalog #SK-4100, Vector Laboratories, Newark, CA) to induce immunostaining visualization. Slides were finally counterstained with Harris Modified Hematoxylin (Catalog #SH26-500D, Fisher Scientific, Hampton, NH) and mounted. The data was quantified with ImageJ software*.*

### Western blot analysis

The heart tissue was homogenized using T-PER Tissue Protein buffer (Thermo Fisher, 78510), supplemented with protease and phosphatase inhibitors (Thermo Fisher, A32959) and proteins were separated by polyacrylamide electrophoresis and transferred onto nitrocellulose membrane as described^16^. After incubation withthe primary antibody, detection was performed using secondary horseradish peroxide–coupled ECL Western Blotting Substrate (Pierce, #32106). The following antibodies were used: rabbit anti-beta actin (Abcam, #ab8227), and rabbit anti–HO-1 (Abcam, # ab13243). The quantification of the data was performed with NIH ImageJ program.

### Oxidative DNA damage analysis

Genomic DNA was isolated using the genomic-tip 20/G (Qiagen,#10223) and DNA Buffer Set (Qiagen, #19060). The detection of apurinic/apyrimidinic (AP) sites was performed using an aldehyde-reactive probe (ARP) kit (Kamiya Biomedical, #DN-002), according to the manufacturer's instructions.

### MDA levels

The protocol for the Oxiselect MDA Adduct Competitive Elisa kit was performed by the procedure described by the manufacturer (STA-832, Cell biolabs).

### Statistical analyses

Statistical analyses were performed using GraphPad Prism 5 (GraphPad Software, Inc., San Diego, CA). Data are expressed as mean ± standard error of the mean (SEM). All parameters were analyzed using one-way ANOVA followed by Holm-Sidak method for multiple comparison.

### Supplementary Information

Supplementary Information.

## Data Availability

All data generated or analyzed during this study are included in this published article and its Supplementary information files. Further inquiries can be directed to the corresponding author. The RNA-seq data have been deposited in the NCBI Sequence Read Archive under BioProject accession number PRJNA953428.
