# Supplementary material for: The lipolysis inhibitor acipimox reverses the cardiac phenotype induced by electronic cigarettes
Source: Sci Rep. 2023 Oct 25;13:18239. doi: 10.1038/s41598-023-44082-x (PMC10600141; doi:10.1038/s41598-023-44082-x)
Supplement: Supplementary file 1 — Supplementary Information. [file 41598_2023_44082_MOESM1_ESM.docx]

**SUPPLEMENTAL MATERIAL**

**
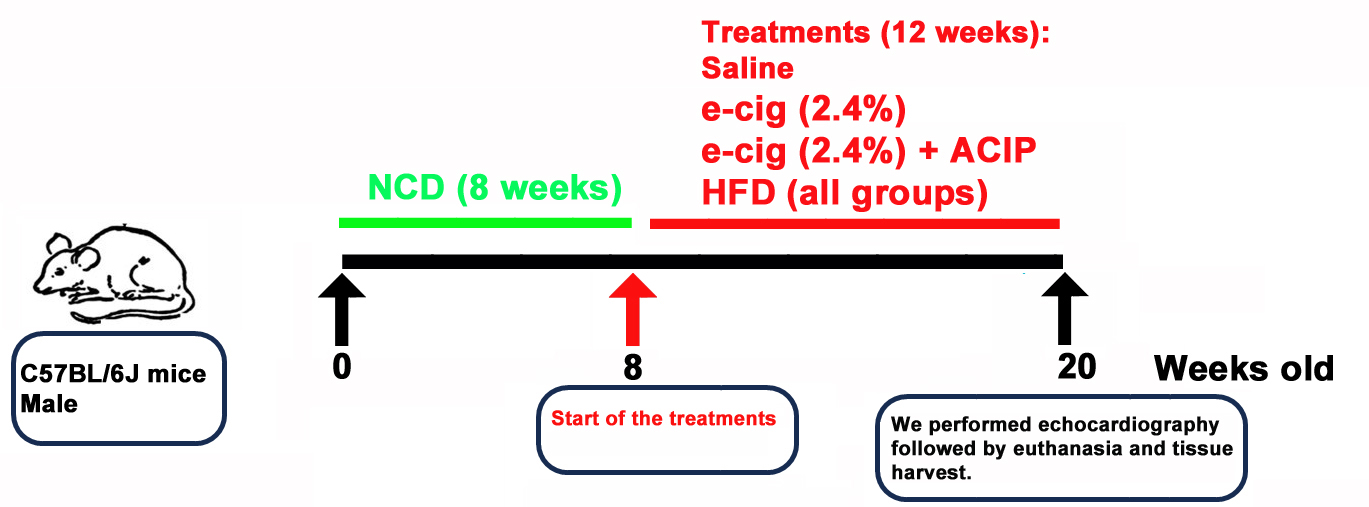
**

**Supplementary Figure S1.** Outline scheme of the mice treatment and experimental protocols.

**
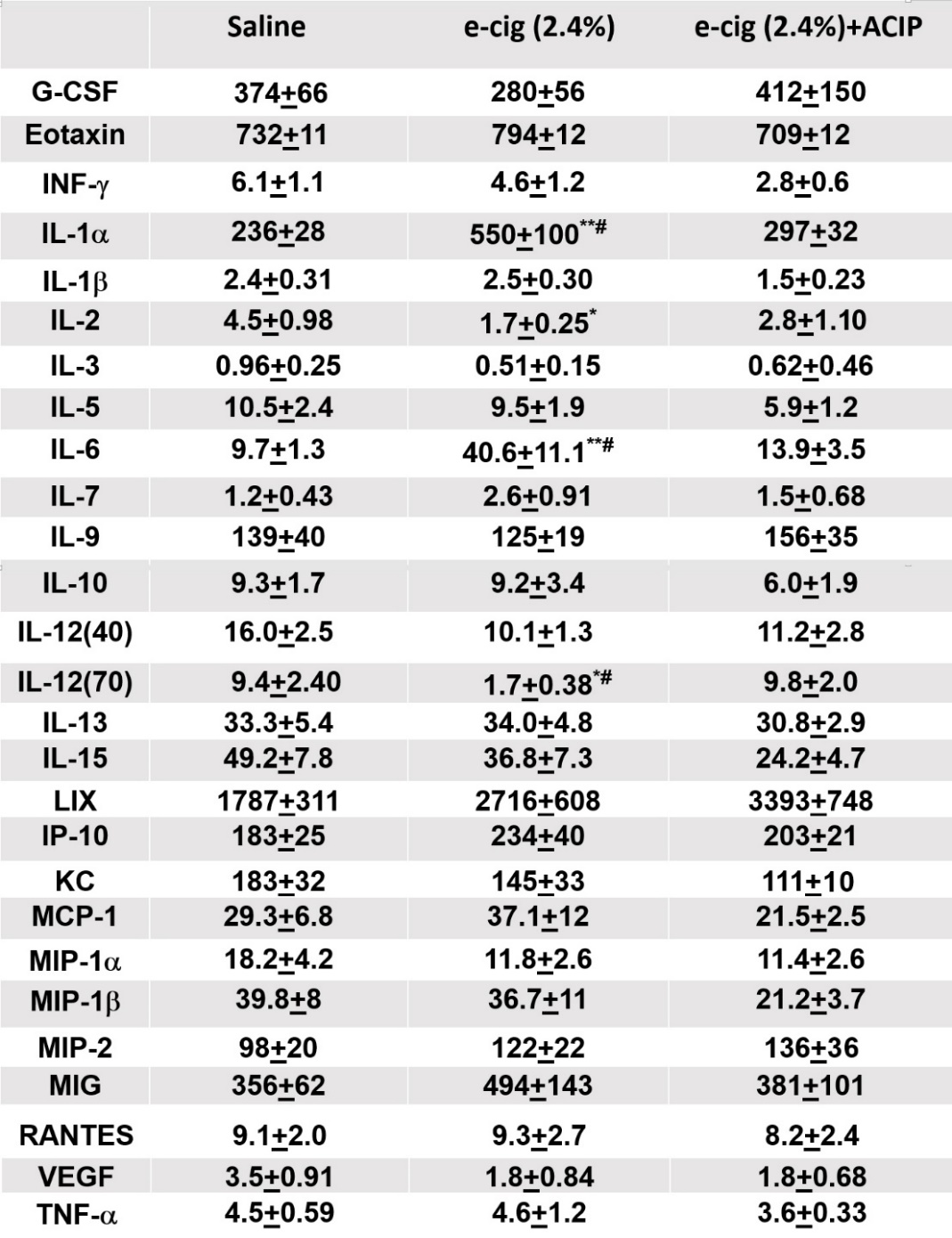
**

**Supplementary Table S1.** Mouse cytokines concentrations in the serum. G-CSF, Granulocyte colony-stimulating factor; INF-γ, Interferon gamma; IL, interleukin; LIX, Lipopolysaccharide-induced CXC chemokine; IP-10, IFN-gamma-inducible protein 10; KC, Keratinocyte chemoattractant; MCP-1, Monocyte chemoattractant protein-1; MIG, monokine induced by interferon gamma; RANTES, regulated on activation, normal T cell expressed and secreted; VEGF, Vascular endothelial growth factors; TNF-α, Tumor necrosis factor alpha. (N=10 per group; saline aerosol vs e-cigarette (2.4%),*P<0.05, **P<0.01; e-cigarette (2.4%) vs e-cigarette (2.4%) + ACIP, ^#^P<0.05 ^##^P<0.01). All values are means ± SEM.


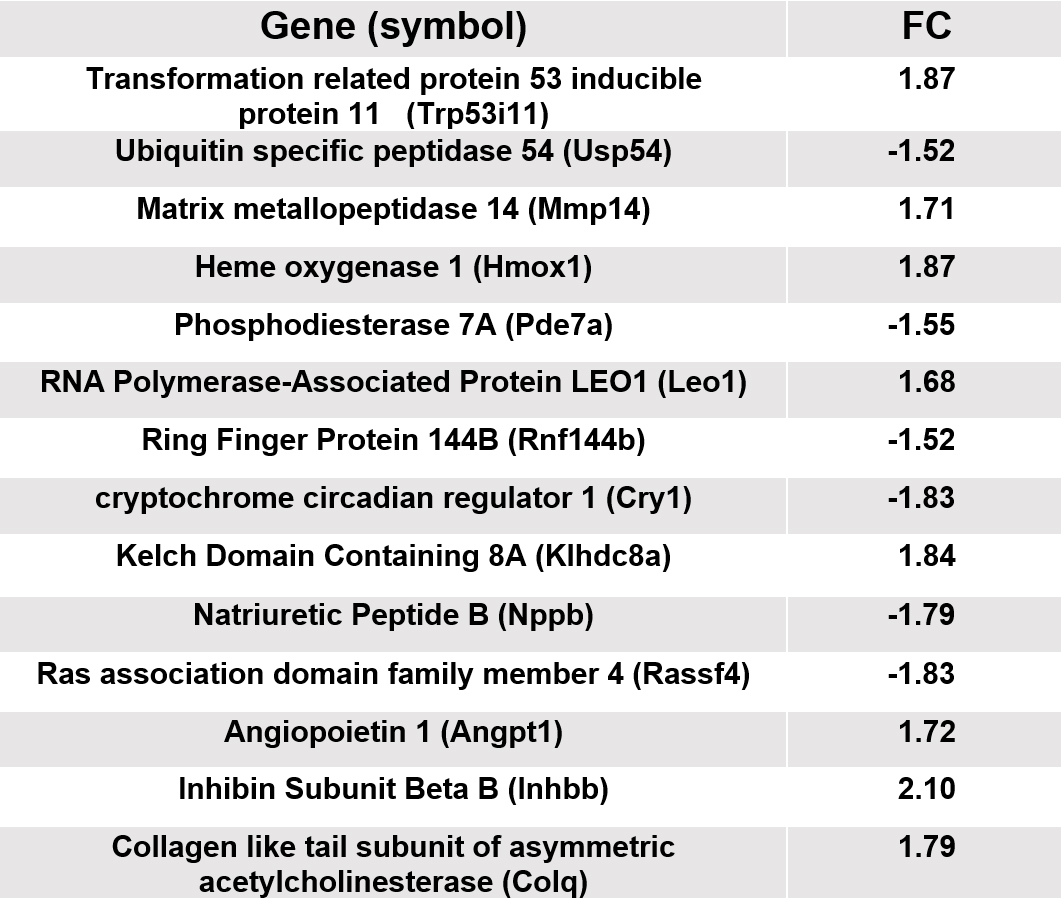


**Supplementary Table S2.** Data generated RNA-seq Analysis, changes in expression from Genes Rescued by Acipimox.

.


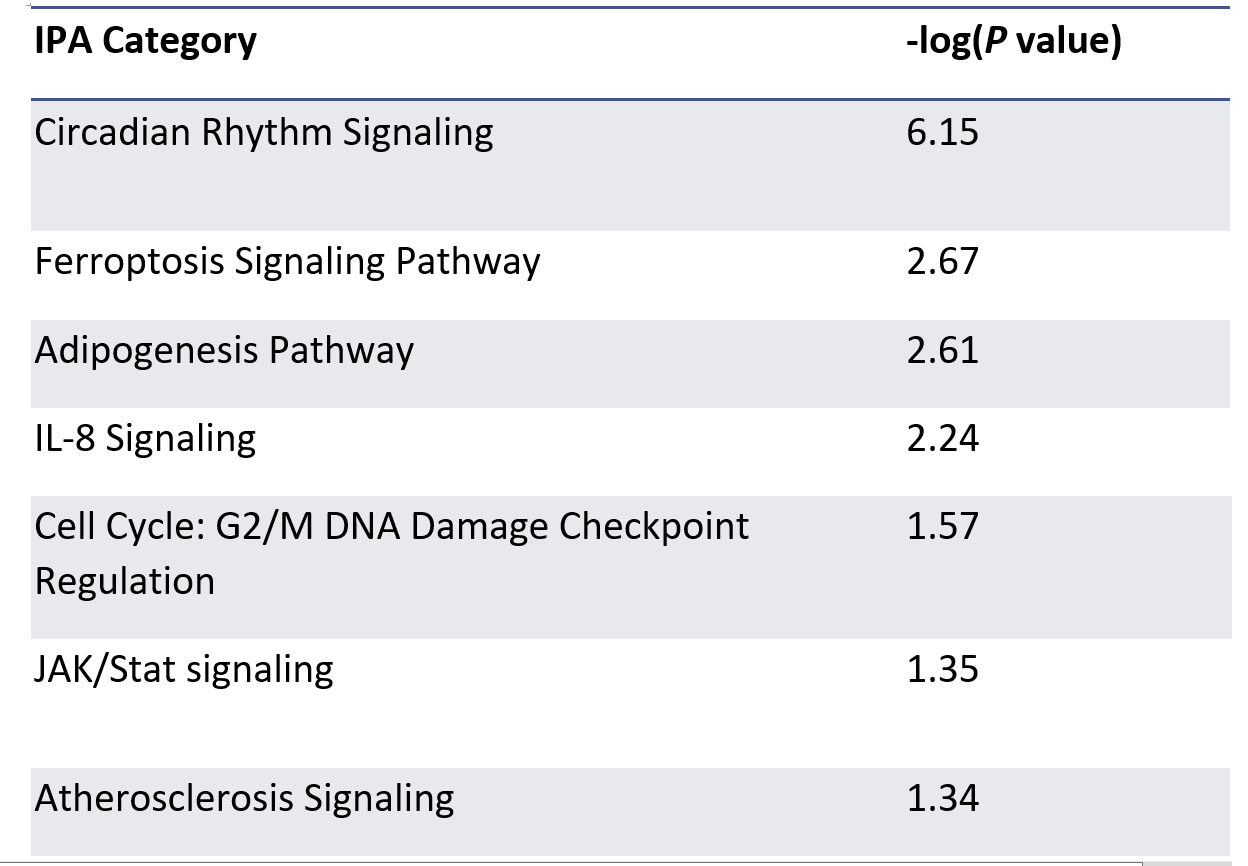


**Supplementary Table S3.** Canonical pathways deregulated in e-cigarette (2.4%)-treated mice relative to control (saline) and e-cigarette plus acipimox.

**
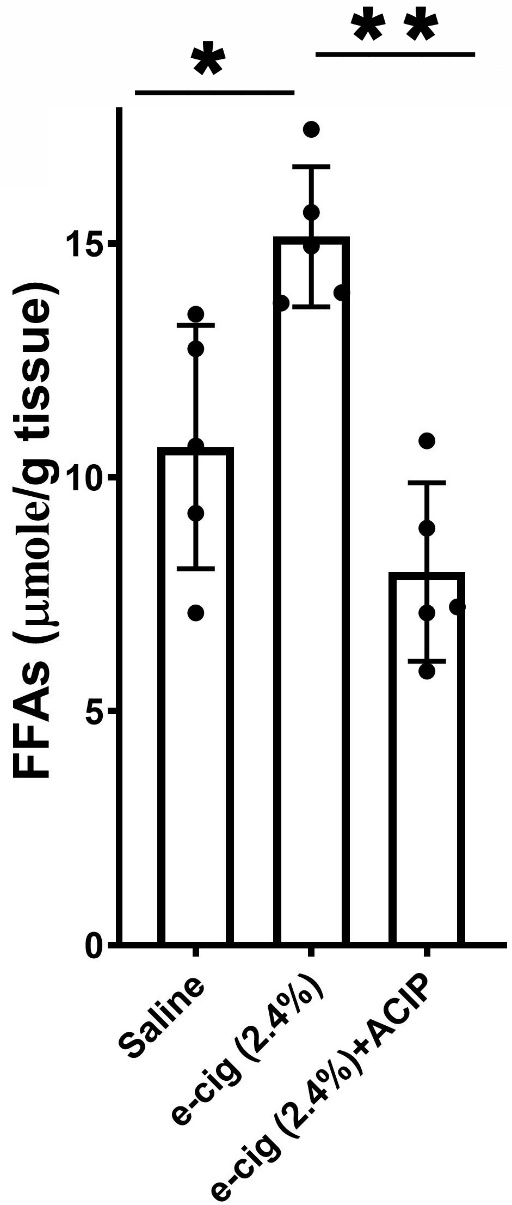
**

**Supplement Fig. S2.** FFAs content in epididymal adipose fat pads in mice exposed to saline, e-cigarette (2.4%), and e-cigarette (2.4%) plus acipimox**.**

**
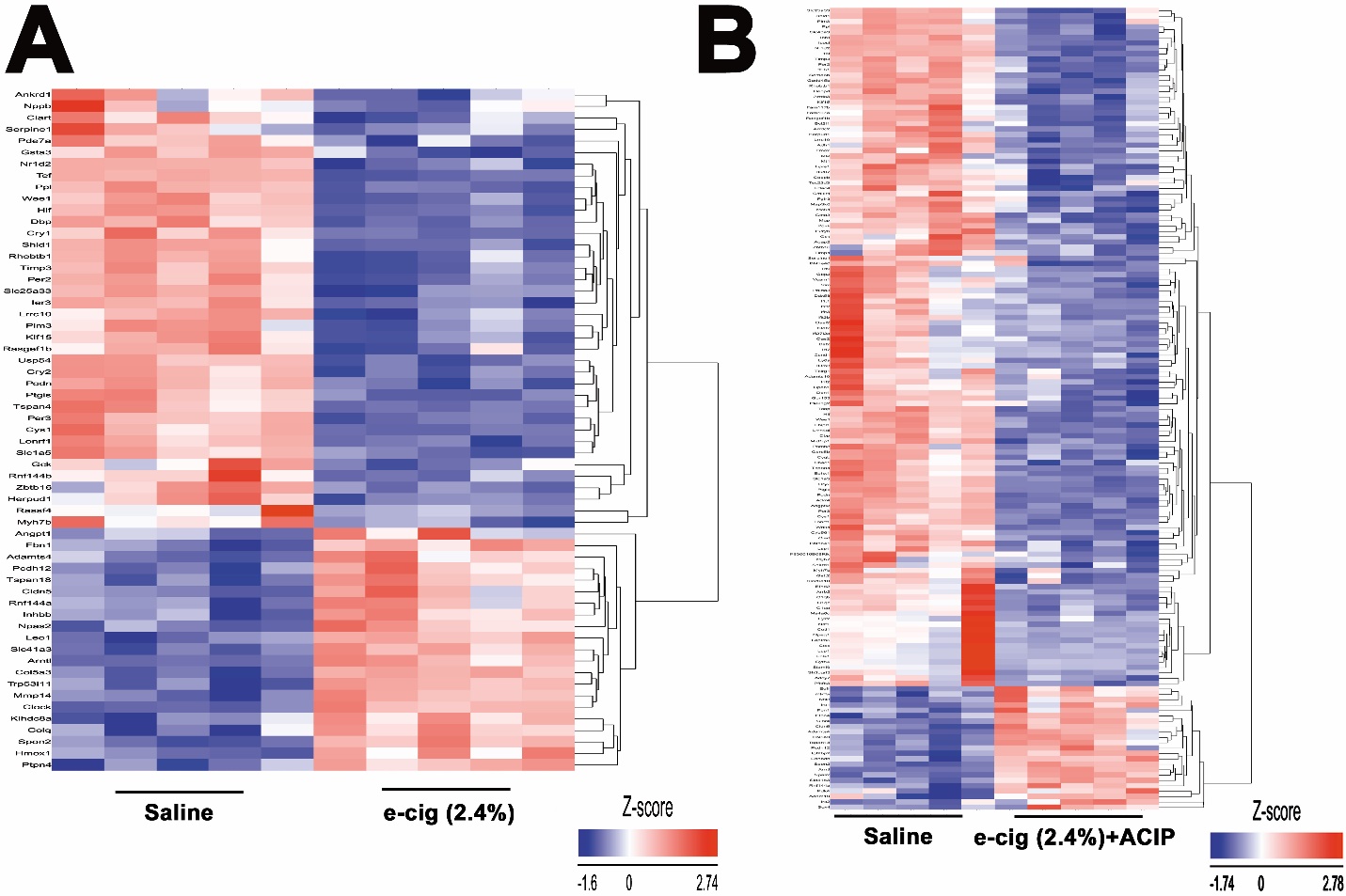
**

**Supplementary Figure S3.** Transcriptomic changes induced by e-cigarette (2,4%) and e-cigarette (2.4%) + ACIP. (A) 2-dimensional hierarchical clustering showing 59 differentially expressed genes between saline and e-cigarette (2.4%). (B) 2-dimensional hierarchical clustering showing 149 differentially expressed genes between saline and e-cigarette (2.4%)+ACIP.

Blot from a nitrocellulose membrane:


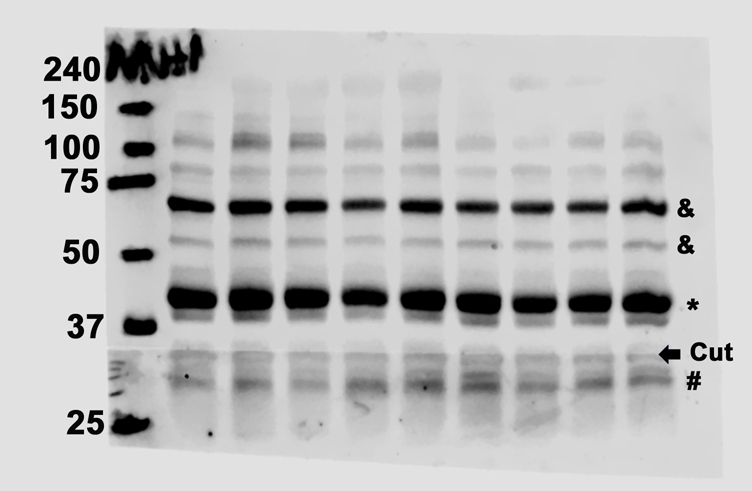


The gel was run until just before the 25KD proteins ran out of the gel. So the membrane can be cut, and HO-1 and actin can be tested simultaneously.

The _*_ symbol marks the bands for actin. The predicted protein molecular weight is 42KD.

The _#_ symbol marks the bands for HO-1. The predicted protein molecular weight is 33KD. But, the observed molecular weight is from 28KD to 33KD.

This membrane was first tested with a different antibody (data not reported). After stripping, the membrane was cut under 37 KD (see black arrow). The membrane was tested simultaneously for actin (top) and HO-1 (bottom). For the development of the western blot, the two pieces of the membrane were put together again. The bands with the “&” symbols are signals not totally stripped for the previous testing.
